# Color tunable pressure sensors based on polymer nanostructured membranes for optofluidic applications

**DOI:** 10.1038/s41598-019-40267-5

**Published:** 2019-03-01

**Authors:** P. Escudero, J. Yeste, C. Pascual-Izarra, R. Villa, M. Alvarez

**Affiliations:** 1 Instituto de Microelectronica de Barcelona (IMB-CNM, CSIC), Campus UAB, 08193 Bellaterra, Barcelona, Spain; 2 ALBA Synchrotron, 08290 Cerdanyola del Valles, Spain; 3 CIBER de Bioengineria, Biomateriales y Nanomedicina (CIBER-BBN), Barcelona, Spain; 4 PhD in Electrical and Telecommunication Engineering, Universitat Autònoma de Barcelona (UAB), Barcelona, Spain

## Abstract

We demonstrate an integrated optical pressure sensing platform for multiplexed optofluidics applications. The sensing platform consists in an array of elastomeric on-side nanostructured membranes -effectively 2D photonic crystal- which present colour shifts in response to mechanical stress that alter their nanostructure characteristical dimensions, pitch or orientation. The photonic membranes are prepared by a simple and cost-effective method based on the infiltration of a 2D colloidal photonic crystal (CPC) with PDMS and their integration with a microfluidic system. We explore the changes in the white light diffraction produced by the nanostructured membranes when varying the pneumatic pressure in the microfluidics channels as a way to achieve a power-free array of pressure sensors that change their reflective colour depending on the bending produced on each sensor. The structural characterization of these membranes was performed by SEM, while the optical properties and the pressure-colour relation were evaluated via UV-Vis reflection spectrometry. Maximum sensitivities of 0.17 kPa^−1^ is obtained when measuring at Littrow configuration (θ_in_ = −θ_out_), and close to the border of the membranes. The reflected colour change with pressure is as well monitorized by using a smartphone camera.

## Introduction

The use of elastomeric materials, such as polydimethylsiloxane (PDMS), for the development of membrane-based optofluidic platforms has been widely reported in the literature^1^. An extended method for tuning membrane based optofluidic devices is through the internal gas or liquid pressure. For example, the fabrication of focus tunable lenses, by changing the membrane deformation, has become an extended method for the development of adaptive optics^2–4^. Different optical detection methods have been applied for measuring the integrated membrane deformation^5–7^. Song *et al*. propose the use of integrated optofluidic membrane interferometers to measure the on-chip microfluidic pressure and flow rate simultaneously, by analysing the interferometric patterns created^8^. Pressure-sensitive paints (PSP), which use luminescence emitted from air pressure-sensitive molecules, have been as well applied for the pressure detection in microfluidics^9^. As an example, pressure sensing inside microfluidic channels bonded to glass chips was achieved by integrating an oxygen sensitive luminescent sensor layer inside of an air-filled cavity^10^.

Other approaches are based on the deformation of embedded optical microstructures (e.g. diffraction gratings), modifying the optical boundary conditions and how the lights interacts with it^11,12^. In these cases, the optical detection schemes are mainly based on the diffraction of a transmitted laser beam and how the diffraction pattern change after deformation^13,14^.

When using white light instead of a laser beam, periodically structured materials exhibit a structural coloration derived from enhanced light scattering in the periodically structured refractive index (photonic crystals). The colour response is especially sensitive to changes in the material structure, which make them very interesting materials able to convert external mechanical stimulus into observable changes of the material colour (mechanochromic)^15–18^. Different techniques has been proposed for the fabrication of flexible mechanochromic photonic crystal for monitoring mechanical deformations^19^. One of the simplest and most extended methods to achieve periodically structured polymeric materials is by using 2D and 3D colloidal assemblies (bottom-up approach), known as colloidal photonic crystals (CPhC). These materials are particularly interesting for the development of visual deformation sensors, including materials swelling or strain^20–22^. Pressure-sensitivity has been as well demonstrated by using inverse opals-type photonic crystals, which produce photonic porous membranes with high sensitivity for force recording^23^, and high sensitive reconfigurable shape-memory polymers^24^. Other approaches are based on top-down processes for the fabrication of the flexible structural coloured systems, including diffraction gratings or photonic crystal slab supporting guided modes resonance (GMR). High refractive index materials are usually required to enhance the diffracted colour or support the propagation of resonance modes, which present serious limitations when using metal or semiconductor layers due to the cracking of this layer during the deformation^25,26^. As an alternative, Karrock *et al*.^27^ reported the fabrication of flexible photonic membranes with quasi-guided modes resonances formed by a refractive index layer of TiO_2_ nanoparticles on a periodically nanostructured surface. With this approach, an estimated limit of detection of 160 Pa was reported by colour imaging of the membrane. High-contrast metastructures (HCMs) made of one single-layer (high-refractive-index material fully surrounded by low-index material) and a periodicity of nearly one wavelength have being as well used for the detection of strain. By using flexible HCM with enhanced -1st diffraction order, Zhu *et al*. reported a large colour change with small deformation^28^.

In this work, we explore the white light diffraction of 2D nanostructured flexible photonic materials to achieve a power-free array of membranes that tune their reflective colour depending on the pneumatic or fluidic pressure inside a microfluidic channel. With this approach, we avoid the use of a high refractive index layer, simplifying the fabrication process and suppressing the effect of this layer on the stiffness of the membrane. 2D inverse colloidal photonic structures were chosen because its colour dependency on the angle of incidence and reflection of the light, and its easy and low cost fabrication (transferable to wafer scale production). At the same time, unlike 1D grating whose color depends on the detection angles (both polar and azimuth) under transverse electric or magnetic polarization, the color of 2D gratings depends only on the polar angle, simplifying the alignment of the system, improving the reproducibility and reducing the error derived from the in-plane position of the device. Elastomeric nanostructured membranes were fabricated by infiltrating a close-packed layer of polystyrene nanoparticles with PDMS. We studied the morphology, mechanical properties and colour tuning of the fabricated membranes when exposed to bi-axial strains by applying pneumatic pressures. We demonstrated as well the suitability of white light interrogation to measure the fluid pressure.

## Platform Design and Working Principle

**Figure 1:**
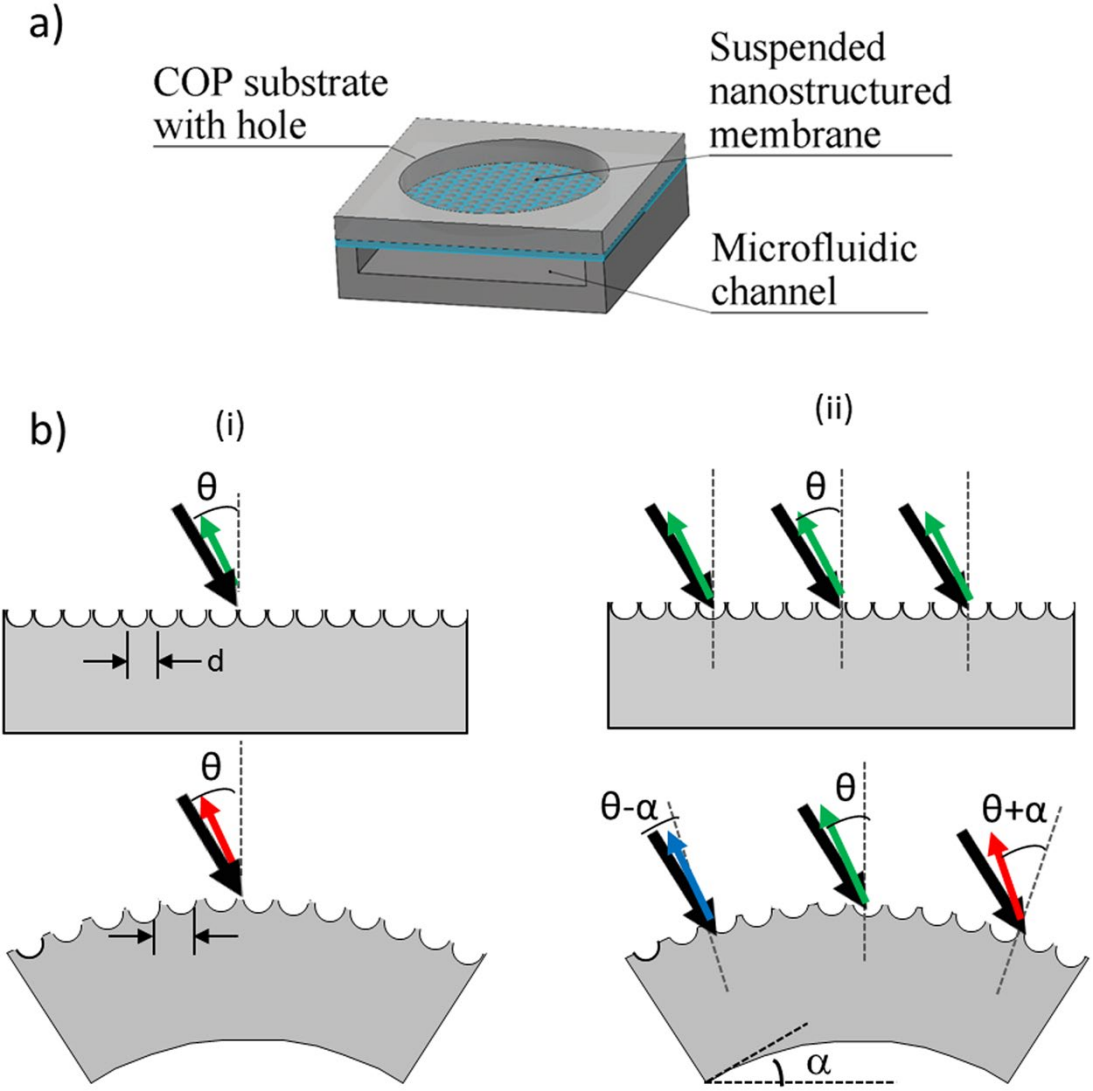
(**a**) Scheme of the device ensemble, and (**b**) schematics representation of the membrane bending and the two possible contributions to the change of the reflective colour.

The colour tuneable pressure sensing platform consists in an array of circular-clamped one-side nanostructured elastomeric (PMDS) membranes, integrated into microfluidics channels, as shown in Fig. 1a. The working principle is based in the reflected colour change of the membranes, when illuminated with white light, under a mechanical stimulation^29^.

A periodic nanostructuration produces a periodic variation in the dielectric function that affects the propagation of photons in certain directions of space, trapping or diffracting light depending on the nanostructure diameter and pitch. The colour reflected by a nanostructured surface when illuminated with a white light source depends on the nanostructure characteristical dimensions, pitch or orientation; a change in any of these parameters, induced by the mechanical deformation, will change the membrane reflected colour. The wavelength change during the membrane deformation has two main possible contributions, as shown in Fig. 1b: (i) an increase in the nanostructures pitch, d, which would produce a red shift on the diffracted wavelength, and (ii) a change in the angle of incidence and reflection of the white light (θ ± α), due to the curvature of the membrane, which would produce a red or blue shift of the wavelength depending on the position of the membrane and the direction of the mechanical deformation.

2D inverse colloidal photonic nanostructuration was selected, instead of 3D photonic structures, for taking advantage of the associated angle change (due to the pressure induced membrane curvature) and maximize the shift in the reflected colour during the out-of-plane membrane deformation. 1D linear grating have the same sensitivity than 2D ones, but with a restriction in the azimuth angle (angle in the membrane plane), being more critical the alignment of the light and the sample, and requiring more expensive fabrication process (top-down) especially for large fabrication areas. For 2D colloidal photonic structures, the incident diffracted light can be calculated from the planar grating equation^30^. There are a number of discrete angles, *α*, for a given groove pitch, *d*, where constructive interference occurs between diffracted light:

1 $$m\lambda =d(sen(\theta )+sen(\beta )),$$

where *m* is the diffractive order, θ is the angle of incidence, β the angle of diffraction and *d*, in the case of a monolayer of colloids, corresponds to $d=\sqrt{3}D/2$ (for a close-packed monolayer) being *D* the sphere diameter. For a specific angle of incident and detection, the membranes will display a specific colour that can be easily measured by visual inspection or by using a camera (smartphone). Looking at Eq. (1), the shift in the reflected colour during the out-of-plane membrane deformation can be maximized by measuring in Littrow configuration, where the angle of incidence is the same as the angle of reflection (θ = −β), reducing the grating diffraction Eq. (1) to $m\lambda =2dsen(\theta )$. For working in the visible range of the spectrum, colloidal spheres of 800 nm and initial incidence angle of 30° were selected.

To increase the membrane deformation for a specific applied pressure, an elastomeric polymer such as PDMS was used for the fabrication of the membranes (low Young’s modulus). The PDMS is a hyperelastic polymer (it supports large deformations without deteriorating), with good biocompatibility, non-porous to the liquids, nontoxic, optically transparent and easily fabricated. Some authors use an incompressible isotropic hyperelactic constitutive model (neo-Hookean) to describe the mechanical response of PDMS membranes^31^. However, for small loadings, the complex hyperelastic model can be replaced by a linear elastic model. The behaviour and sensitivity of elastomeric membranes present a large dependency on the membrane thickness, *t*. In the case of thick membranes (maximum deflection is much smaller than its thickness) the shape of the deflection is determined by the bending moments acting especially at the rim where the membrane is clamped. When the membrane is deflected, its neutral axis is stretched, generating some stress according to Hooke’s law. For thin membranes (maximum deflection is larger than its thickness), the bending moments could be neglected, and the membrane deformation adopts a parabolic profile. A thick membrane would change its behaviour to a thin one as the pressure rises and the deflection is increased. In general, both bending moments and stress could affect the membrane behaviour depending on the pressure applied. The deflection of elastomeric circular-clamped membrane subjected to a uniform pressure *P* is given by Eq. (2) ^32^:

2 $$P=\frac{16{t}^{3}E}{3(1-{\nu }^{2}){a}^{4}}{w}_{0}+4\frac{t{\sigma }_{0}}{{a}^{2}}{w}_{0}+2.43\frac{Et}{(1-{\nu }^{2}){a}^{4}}{w}_{0}^{3}$$

where *w*_0_ is the maximum deflection at the centre of the membrane, ν is the Poisson coefficient, E is the Young’s modules and *a* is the radio of the membrane. The three terms in Eq. (2) describe the contribution of the bending moments, residual stress σ_0_, and stress due to straining of the neutral fibre, respectively. Bending moments and residual stress show a linear interrelationship between pressure change and membrane deflection, while the stress due to straining contributes with its third power. This membrane behaviour suggest that for detecting low pressure changes, in a low pressure range, the governing parameter will be the change in the curvature of the membrane, discarding the effect of the neutral fibre strain (no change in the thickness of the membrane).

## Device Fabrication

The pressure sensing platform was fabricated using a multilayer approach. An initial one-side nanostructured elastomeric thin layer was prepared over a hard substrate, which define the thickness of the suspended membranes. Next, the elastomeric photonic thin layer was sandwiched between two substrates: one with perforated holes to define the suspended membranes dimensions (1.5 mm in diameter), and another one with straight microfluidics channels (as previously shown in Fig. 1a).

### Elastomeric photonic layer

The on-side nanostructured elastomeric layer was prepared using 2D CPhC as master moulds. The 2D CPhC were prepared by the air-liquid interface method. Surface tension, evaporation temperature and percentage of surfactant in nanoparticles solution are essential variables in this process to achieve a high order close-packed monolayer. An aqueous suspension of 800 nm polystyrene nanoparticles was diluted in ethanol, to reduce its surface tension, to form a 6 wt% mixture. A nanoparticle monolayer was created at the air-water interface by releasing 200 µl of the solution over a partially immersed hydrophilic glass slide with a tilt angle of 20°. A hydrophilic glass slide, previously immersed on the water volume, was used to collect the nanoparticles monolayer by removing the water carefully. Finally, the assembled nanoparticles were dried during 30 minutes at 80 °C to evaporate the water and improve the crystallization. A thin layer of PDMS (10:1 ratio) was spin coated over the dried particles monolayer, and immediately cured at 100 °C for 1 hour. The PDMS thickness, and therefore the membranes thickness, depends on the spin coating speed. In this case we prepared a PDMS thin layer of 50 ± 2 µm. During the coating process the PDMS fills the interparticle gaps while the nanoparticles remain stuck to the substrate. When peeled off from the substrate, the PDMS left an inverse shape into the cured PDMS. More information about the 2D photonic crystal mould fabricated using 800 nm nanoparticles can be found in the Supplementary Information (Fig. S1). The glass slide with the assembled nanoparticles can be reused as a master mould.

### Packaging/integration

Before peeling-off the one-side nanostructured PDMS layer, a PDMS substrate with the microfluidic network was bonded to it. The ensemble was then peeled-off from the glass substrate, and the nanostructured side was bonded to a COP substrate with perforated holes that define the array of membranes. The membrane shape and size was designed using a computer aided design program (Vcarve). The PDMS microfluidic network were fabricated by master moulding, using SU8 master moulds fabricated by standard photolithographic process, and bonded to the nanostructured membrane by oxygen plasma treatment. The COP substrates were prepared using rapid prototyping techniques (laser or CNC micromachining), and silanized with AMPTS, previous to the bonding with the oxygen plasma treated photonic membrane.

## Characterization and Discussion

### Morphological characterization of the membranes

**Figure 2:**
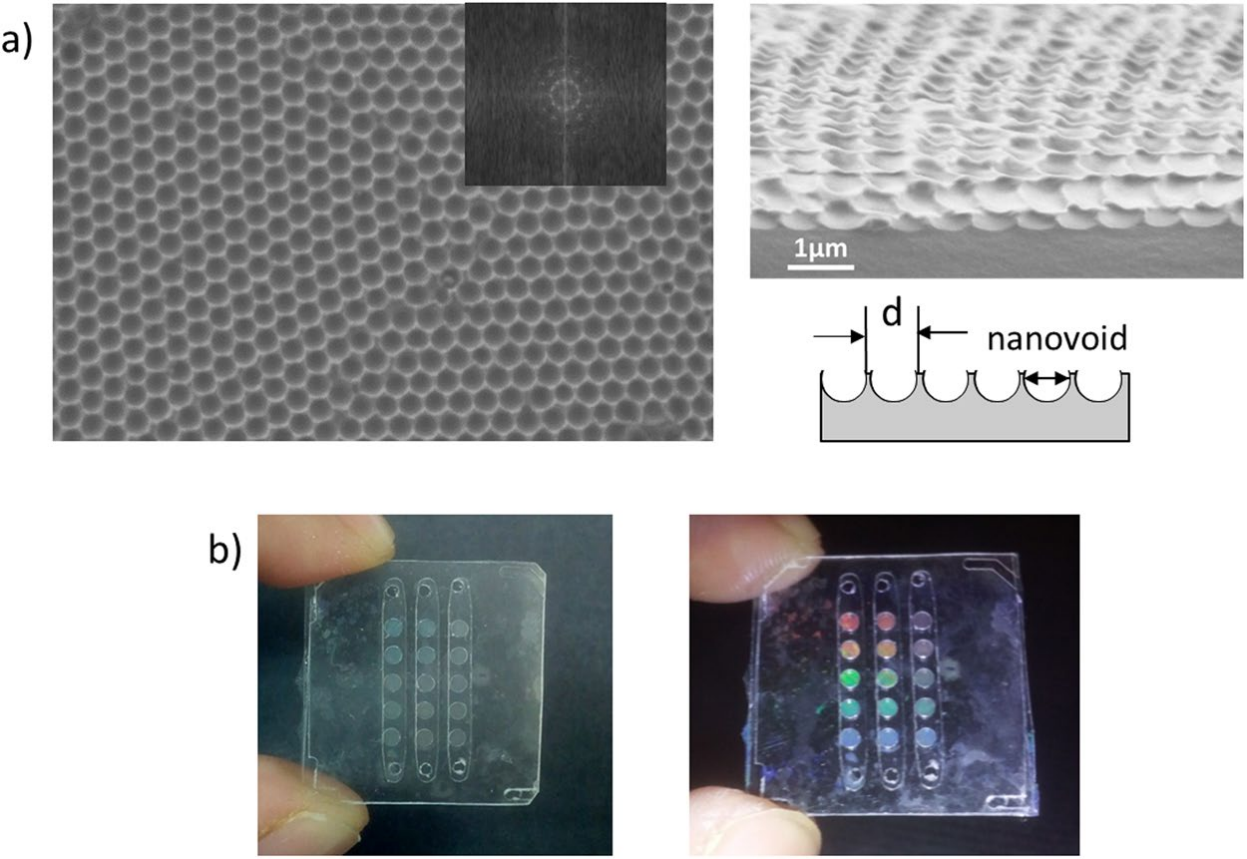
(**a**) SEM images (top view and in angle) of the voids leaved on the PDMS. (**b**) Photograph of the final integrated device at two different angles.

During the fabrication of the elastomeric photonic membranes, scanning electron microscopy (SEM) was used to evaluate the packaging of the self-assembled latex nanoparticles, the PDMS infiltration, and the voids left after the peeling process. Figure 2a shows the high ordered 2D inverse shape left into the PDMS after the peeling process which will work as diffractive surface with the ability to separate the different wavelengths of light. The bowl-shaped nanovoids have a mean diameter of 691 ± 21 nm, calculated from SEM images by using ImageJ software (National Institutes of Health, USA) and a height of about 320 nm. More information can be found in the Supplementary Information (Fig. S2). These nanovoids are 7% smaller than the nanoparticles used to prepare it. This size discrepancy can be attributed to the PDMS expansion after the peeling process. The 2D PDMS nanostructure pitch is 745 ± 78 nm, in accordance with the PS nanoparticles size used. The FFT of the SEM images was calculated as indicator of the crystal order. The FFT of the SEM image is shown in Fig. 2a inset, were the bright spots demonstrate the high crystal order achieved.

After the integration and packaging process, the final device is completely transparent, while the membranes exhibit a strong coloration at specific angles of diffraction, as shown in Fig. 2b. The lack of colour of the device out of the membranes is due to the squash of the nanostructures when bonded to the plastic substrate that supports them.

### Mechanical characterization of the membranes

**Figure 3:**
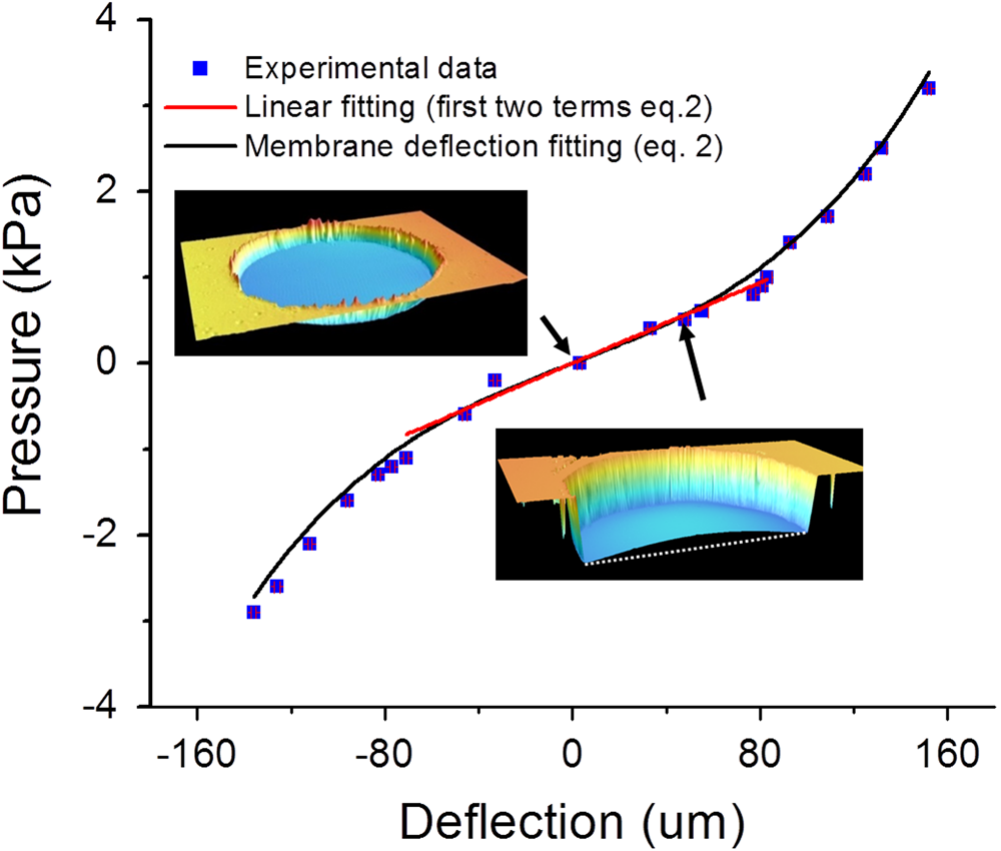
Pressure-deflection curve obtained by confocal microscopy for a 50 µm thickness nanostructured membrane, and fitting of the experimental values to Eq. (2) (in black, R = 0.989) and to the first two terms of Eq. (2) (in red, R = 0.965).

The membranes behaviour under pneumatic deformations where experimentally characterized via confocal microscopy. Figure 3 shows the measured deflection at the centre of the membrane for a range of pneumatic pressures of ±3 kPa, and the fitting to the theoretical membrane deflection model described by Eq. (1).

For a pressure range of ±3 KPa, Eq. (2) fits well the experimental data, suggesting that the fabricated suspended membranes behave as a thin membrane for this range. Young’s modulus and residual stress values of 1 MPa and 0.02 MPa, respectively, were found from the fitting when considering a Poisson ratio of 0.5, membrane thickness of 50 µm and membrane radio of 0.75 mm, which are in the range of the reported values for PDMS membranes^33^. In the low pressure range, between −1 and 1 kPa, the measured membrane deflection is smaller or in the order of the membrane thickness. In this case, the experimental data can be fitted to the first two terms of Eq. (2). For the same fixed conditions than before, the found Young’s modulus and residual stress values were 1.3 MPa and 0.02 MPa, respectively, in accordance with the values found when fitting to the whole equation. This behaviour suggests that for small loadings the use of the linear elastic model is suitable.

### Pressure sensitivity analysis by UV-Vis spectroscopy

A customized UV/Vis set-up and custom software based in Python language^34^ was developed to simultaneously monitor the spectra, find the wavelength peak and measure the pressure. The pressure sensing platform was mounted on a two dimensional lineal stage to select the membrane and the measurement position. Out-of-plane deformations (bi-axial strains) were produced in the membranes by a controlled pneumatic pressure injection into the microchannels. A pressure sensor (DP-101A-E-P), connected at the microchannel output, was used to measure the applied pressure. A syringe was used to control the pressure.

**Figure 4:**
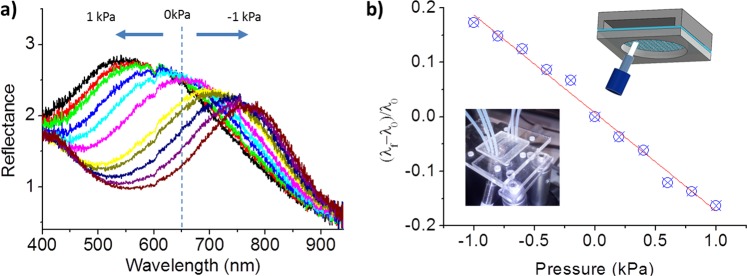
(**a**) Colour response of a single membrane for pressure ranges between −1 kPa and 1 kPa. (**b**) Calibration curve for positive and negative pressures (linear fitting with slope = 0.17 0.17 KPa^−1^ and R = 0.988) and insets: photograph and scheme of the fabricated pressure sensing platform and experimental set-up for UV-Vis spectrometry.

UV/Vis reflection spectrometry was used to characterize the colour response of the photonic membranes by applying incremental constant pressures. The diffracted colour was measured by using a bundle reflection probe in a Littrow configuration^35^, with an angle between the probe and the normal to the membrane of 30°. At this configuration, the expected theoretical diffraction wavelength considering the mean pitch value of 745 nm obtained from the SEM images is 645 nm. The experimentally measured diffracted colour of flat membranes was 647 ± 4 nm, as shown in Fig. 4a, in accordance with the expected theoretical value. If the membranes are initially buckled, we can expect a change in the reflection wavelength depending on the position of focus, due to the dependency of the angle of incidence and reflection of the white light with the curvature of the membrane, as previously shown in Fig. 1b. A scan of the probe along the membranes shows that the membranes are initially flat and do not suffer from initial bending. Similarly, the reflected measured wavelength would increase or decrease for positive pressures, depending on the position of focus in the membrane, and vice versa for negative pressures.

The colour response of the membranes under pressure deformations were analysed for a low pressure range of ±1 kPa. The obtained spectral peaks when focusing close to the left border of the membrane are shown in Fig. 4a. The small variations observed on the spectral intensity are due to changes in the distance between the reflection probe and the membrane during the deformation process: negative pressures increases the probe-membrane distance (decreasing of the light collected by the reflection probe), while positive pressures reduce this distance. The photonic membranes show a linear behaviour (see Fig. 4b) for the range of applied pressures (linear fitting with R = 0988). Maximum experimental wavelength change of 117 nm was measured when applying 1 kPa pressure. This maximum wavelength change is different depending on the region of focus, being higher when focusing close to the border of the membrane where the angle changes are larger (see Fig. S3 in Supplementary Information). This result suggest that the main contributor to the diffracted colour shift is the change of the membrane curvature (change in the effective angle of incident and reflection), as described in Fig. 2b (ii). For a fixed position of the fibre (close to the border), the devices show a long-term stability (see Fig. S4 in the Supplementary Information).

In an independent series of experiments, we measured the colour change produced when focusing in a flat nanostructured PDMS by changing the angle of incidence (see Fig. S5 in Supplementary Information). In this case, a linear colour change that fits the Eq. (1) in Littrow configuration for a particle diameter of 790 nm was obtained, which matches the values calculated from the SEM images. A mean shift of around 20 nm per degree was found.

Analysing the membrane profile, the vertical displacement at the centre of the membrane is 83 µm when applying 1 kPa pressure (obtained from the confocal measurements from Fig. 3), which corresponds to an angle change of 6.5° measured close to the border of the membrane. Considering a wavelength change of 20 nm/°, the estimated change in the reflected wavelength is 120 nm when applying a pressure of 1 kPa, which is very close to the experimentally measured value (117 nm/kPa). This confirms that the contribution from the nanostructure deformation is negligible respect to the contribution from the angle change, for the low pressure regime.

The pressure sensitivity of the pressure sensor, S, can be defined as the slope of the relative wavelength change-pressure curve in Fig. 4b: $S=\delta ({\rm{\Delta }}\lambda /{\lambda }_{0})/\delta P$, where ${\rm{\Delta }}\lambda ={\lambda }_{f}-{\lambda }_{0}$, λ_f_ and λ_0_ denote the wavelength with the applied pressure and the wavelength at cero pressure, respectively, and P denotes the applied pressure. The sensor shows a sensitivity of 0.17 KPa^−1^ for the low pressure regime (−1 to 1 KPa). Higher sensitivities can be achieved by adjusting the diameter and thickness of the membrane. Considering only the elastic model (first term in Eq. (2)), larger deformations of the membrane would be obtained by increasing the ratio *a*^4^/*t*^3^.

With this channel configuration we as well assessed the detection of the in-channel flow rates for different solutions. The pressure-flow characteristic of Newtonian incompressible fluid flow through a rigid rectangular microchannel with high aspect ratio (length/width) is given by Poisseuille’s law: ΔP = 12 µQ/h^3^w, where ΔP is the pressure drop along the channel, Q is the flow rate, L and w are the length and width of the channel, respectively, and µ is the viscosity of the fluid. This relation shows the dependency of the pressure with the viscosity of the solution inside the channel. However, because of the low aspect ratio of our channels and the deformation of the membranes when injecting the flow, the relation between the pressure drop along the channel and the flow rate is not-linear, unlike the rigid channel. Several authors have propose different models (depending on the channels dimensions) to explain the non-linear behavior of microfluidics channels with flexible walls, demonstrating smaller pressure drop for a specific flow velocity than in the case of rigid channels^36^. For example, Gervais *et al*.’s model for flexible microchannel demonstrated that the flow rate is a quartic polynomial of the pressure drop^37^, while for Christov *et al*. the flow rate is a cubic polynomial of the pressure^38^.

**Figure 5:**
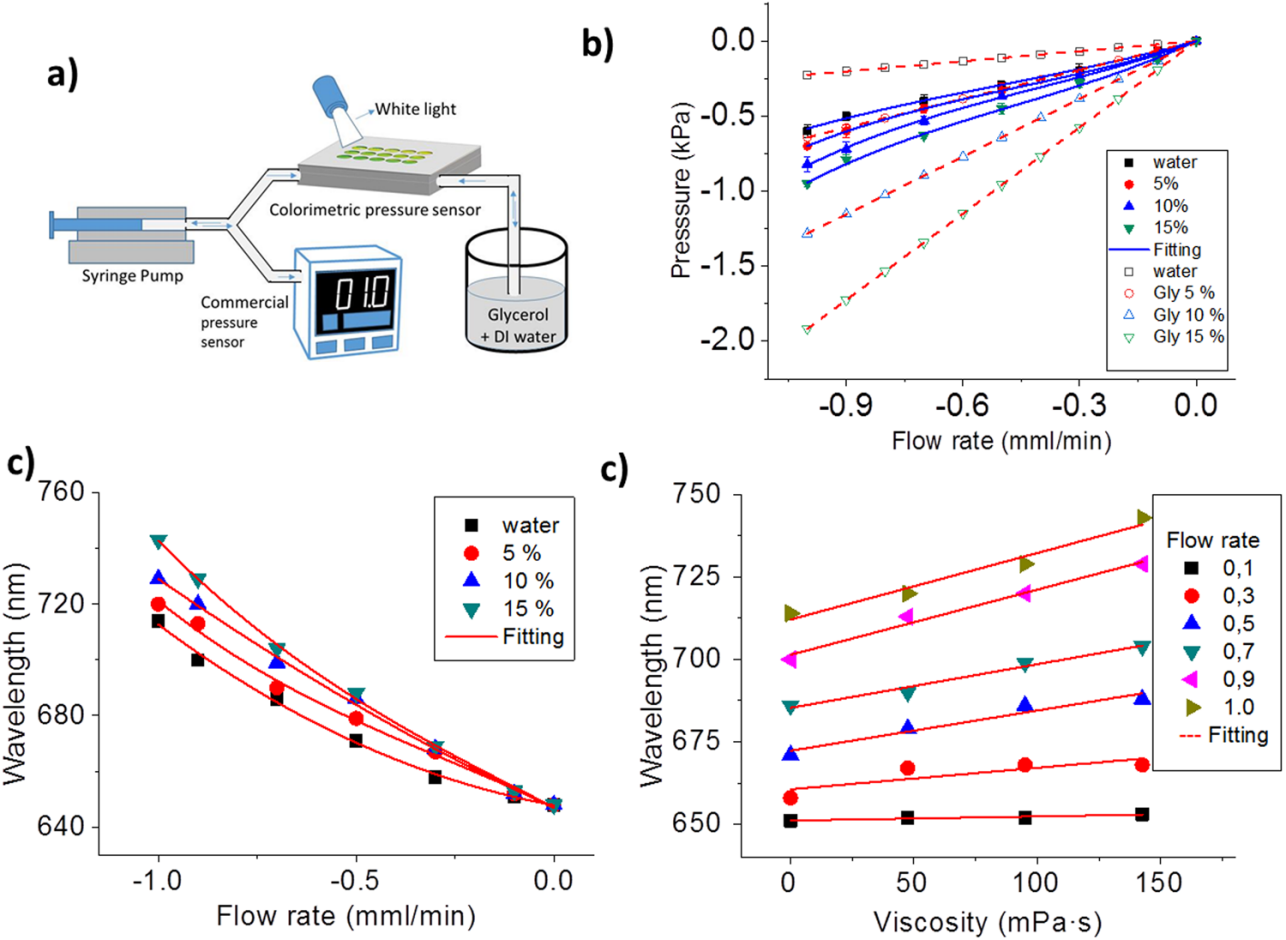
(**a**) Scheme of the set-up configuration. (**b**) Pressure-flow rate curve: experimental (continuous line) and theoretical expected values (dot lines) from Poisseuille’s law (rigid channels). (**b**) Wavelength-flow rate calibration curve for solutions with different percentage of glycerol (four different viscosities). (**c**) Wavelength dependency with the fluid viscosity for different flow rates.

Experimentally, the chip was connected to a high precision syringe pump, which pumped the solutions through the channel at rates from 0 to 1 ml min^−1^. A scheme of the set-up configuration is shown in Fig. 5a, where the commercial pressure sensor used to calibrate the system was connected between the syringe pump and the chip. Three water-glycerol solutions with different dynamic viscosity and density were prepared. The generated pressure increased non-linearly with the flow rate, and with the solution viscosity, as expected considering that the array of membranes inside the channel deform when applying a flow rate (Fig. 5b). The experimentally measured pressure is much lower than the expected from a rigid channel (dot curves in Fig. 5b), in accordance with the behaviour of flexible channels^36–38^ even though this models do not properly fit our experimental behaviour (data do not shown) due to the added complexity of having an array of flexible membranes along the rigid channel (instead of a continuous flexible wall). This non-linear behaviour is very clear when measuring the corresponding wavelength shift produced when increasing the flow rate and the fluid viscosity, due to the induced pressure change and the resultant deformation of the membrane (Fig. 5b). In fact, the induced wavelength change increases with the flow rate following a third order polynomial. For flow rates larger than 0.3 ml/mm, which would produce pressures changes higher than ±1 kPa, the wavelength-flow rate characteristic curve would exhibit a more complex relation due to the additional non-linear mechanical response of the membranes. For a specific flow rate, the wavelength change increases linearly with the fluid viscosity, and the viscous sensitivity increases for larger flow rates (shown in Fig. 5c), as expected from Poisseuille’s law and compliant PMDS channels models (higher viscosity produce higher pressure changes, and therefore in our case, higher membrane deformation).

### Image evaluation

#### Smartphone-based set-up for image acquisition

A prototype accessory was developed to convert a smartphone into a real-time imaging platform based on white light interrogation mechanism. The accessory was designed using a computer aided design program (Vcarve) and the different parts were fabricated out of black PMMA by laser engraving (Epilog Laser Mini 24). The accessory fixes the angle between the sensing platform and the smartphone to 30°. The smartphone flash LED is used as light source, illuminating the whole platform. Light reflecting off the sensor surface is focused on the smartphone’s camera sensor by an external plastic imaging lens (focal length = 24 mm).

**Figure 6:**
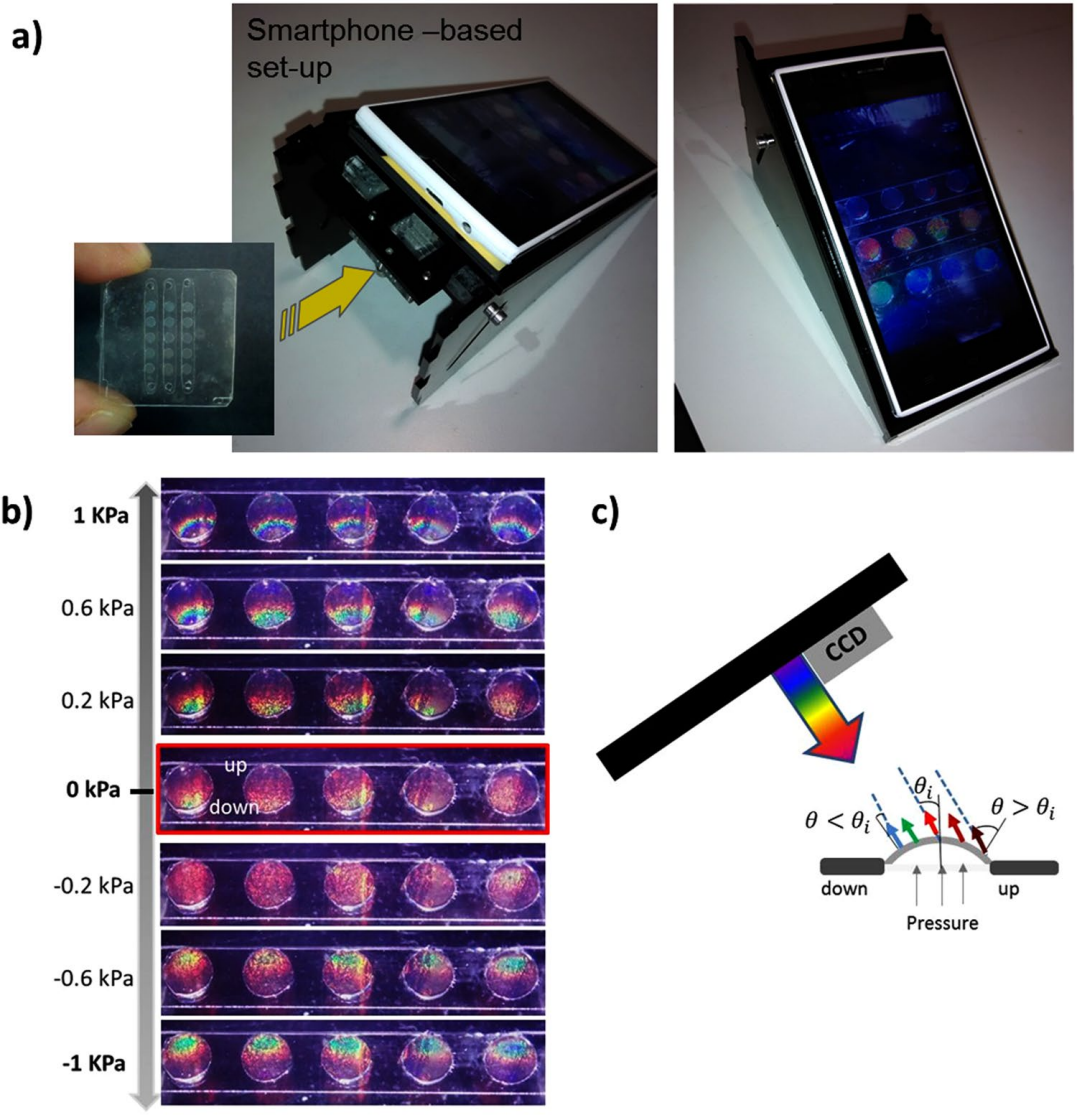
(**a**) Smartphone-based image acquisition set-up. (**b**) Sequential images acquired for a range of applied pressures (−1 kPa to 1 kPa). (**c**) Image set-up scheme showing the angle change during membrane deformation and the associated colour pattern along the membrane profile.

As a proof of concept, the chromatic response of the membranes under pressure stimulation was also evaluated by image colour analysis. Figure 6a shows a smartphone-based set-up developed to that effect. This methodology allows the simultaneous analysis of the whole array of membranes without the necessity of complex instrumentation. When applying an external pressure, the bending of the membrane produced a gradient of colours along the membrane (Fig. 6b), due to the change on the effective angle of incidence and reflection of the light along the membrane profile, as previously observed by UV-Vis spectrometry.

Figure 6b shows a sequence of images of a single array of membranes inside a channel when applying different pressures. The membranes show a large colour change when applying low pressures (−1 to 1 kPa), displaying colours strips (blue, green yellow and red) that correspond to the reflected light at different parts of the membranes with different local surface angle as a consequence of the curvature, as schematized in Fig. 6c. A blue-shift and red-shift is shown depending on the side of the membrane (named as up or down in Fig. 6b) respect to the central part, where there is no effect of the curvature on the angle of incidence or reflection. Positive pressures produce an inflation of the membranes, as shown in Fig. 6c. In this case, the bottom half of the membrane have incidence and reflected angles (respect to the normal to the membrane surface) that are smaller than the initial angle of 30° (respect to the flat membrane), which produce a blue-shift of the reflected colour. In opposition, the upper half of the membrane presents an increase in the incident and reflected angle, producing a shift towards infrared values which are filtered-out in our conventional phone camera. When applying negative pressures the behaviour is the contrary, showing the blue-shift in the upper half of the membrane. It can be observed that pressures changes of 0.2 kPa are clearly distinguishable by naked eye. As next step, we are developing the automated computer analysis of the colour in real time.

## Conclusions

We demonstrated a label-free and power-free array of colour tunable pressure sensors based on flexible nanostructured suspended membranes for multiplexed optofluidics applications. Our approach based on white light interrogation to measure the membranes bending is successfully demonstrated by using both UV-vis spectrometry and image analysis. The fabricated optical pressure sensing platform displays a specific reflective colour for each sensor depending on the pressure-induced bending. The expected gradient of colours along the membrane was observed by image evaluation, depending on the pressure applied, and was found to agree with the wavelength dependency on the position of focus when using UV-vis spectrometry. A simple and cost-effective method was used for the fabrication of the suspended photonic membranes. The platform shows a sensitivity of 0.17 KPa^−1^ for the detection of low pneumatic or fluid pressures (in a range between −1 and 1 kPa), similar to others works with more complex fabrication processes. In addition, a higher sensitivity could be achieved by adjusting the membrane size and the PDMS thickness. Future plans include the development of image analysis software for the automatic evaluation of the membranes colour change in real time.

## Materials and Methods

### Chemicals

800 nm latex nanoparticles (10% w/v), and 3-(aminopropyl)triethoxysilane (AMPTS, 99%) were purchased from Sigma-Aldrich. 1H,1H,2H,2H-perfluorooctyl-trichlorosilane (PFTS, >97%) was obtained from ABCR (GmbH Germany). Sylgar 184 PDMS elastomer kit (Down Corning) was purchased from Ellsworth Adhesives (Spain), and Glycerol (99,5%) from Scharlau Chemie SA.

### Structural characterization

All scanning electron microscopy (SEM) images were obtained using a Auriga-40 microscope from Carl Zeiss, with an accelerating voltage of 1–2 kV.

### Characterization of membrane deformation with pressure

Membranes behaviour during pressure deformations where experimentally characterized via confocal microscopy and UV/Vis spectrometry. A confocal microscope (pLµ NEOX 3D Optical Profiler, Sensofar) was used to determine the maximum deflection at the centre of the membrane. A Flame UV/Vis spectrometer (Ocean Optics) connected to a bundle reflection probe (which emits white light from a central fibre and collects the back-diffracted light with a surrounding set of six fibres; Thorlabs Inc.), in a Littrow configuration, was used to measure the colour response of the photonic membranes. The spot diameter of the incident light was about 450 µm, covering 1/3 of the membrane. The experimental angle between the probe and the normal to the membrane was θ = 30°.

## Supplementary information

Color tunable pressure sensors based on polymer nanostructured membranes for optofluidic applications

## Data Availability

The datasets generated during and/or analysed during the current study are available from the corresponding author on reasonable request.
