# Supplementary material for: Color tunable pressure sensors based on polymer nanostructured membranes for optofluidic applications
Source: Sci Rep. 2019 Mar 1;9:3259. doi: 10.1038/s41598-019-40267-5 (PMC6397196; doi:10.1038/s41598-019-40267-5)
Supplement: Supplementary file 1 — Color tunable pressure sensors based on polymer nanostructured membranes for optofluidic applications [file 41598_2019_40267_MOESM1_ESM.pdf]

SUPPLEMENTARY INFORMATION

Color tunable pressure sensors based on polymer nanostructured membranes for optofluidic applications

P. Escudero[a,d], J. Yeste[a], C. Pascual-Izarra[b], R. Villa[a,c] and M. Alvarez[a,*]

[a] Instituto de Microelectronica de Barcelona (IMB-CNM, CSIC), Campus UAB, 08193 Bellaterra, Barcelona, Spain.
[b] ALBA Synchrotron, 08290 Cerdanyola del Valles, Spain
[c] Biomedical Research Networking Center in Bioengineering, Biomaterials and Nanomedicine (CIBER-BBN), Zaragoza, Spain
[d] PhD in Electrical and Telecommunication Engineering, Universitat Autònoma de Barcelona (UAB)

*corresponding author: mar.alvarez@imb-cnm.csic.es

1. Photonic membrane fabrication.

Fig. S1 shows the fabrication steps follow for the development of the polymeric photonic membranes. The fabrication is grounded in the production of two dimensional photonic crystals based in the assembly of PS colloidal nanoparticles (2D CPhC). The 2D CPhC were prepared by the air-liquid interface method. A nanoparticle monolayer was created at the air-water interface by releasing 200 µl of the solution over a partially immersed hydrophilic glass slide with a tilt angle of 20°. A hydrophilic glass slide, previously immersed on the water volume, was used to collect the nanoparticles monolayer by removing the water carefully (as shown in Fig. S1 (i)). The assembled nanoparticles were dried during 30 minutes at 80°C to evaporate the water and improve the crystallization, to achieve 2D CPhC as shown in Fig. S1 (i). In a second step, the 2D CPhC was spin coated with PDMS. And finally, in a third step, the PDMS was peel-off from the substrate, leaving an inverse pattern on the PDMS, as shown in Fig. S1 (iii).

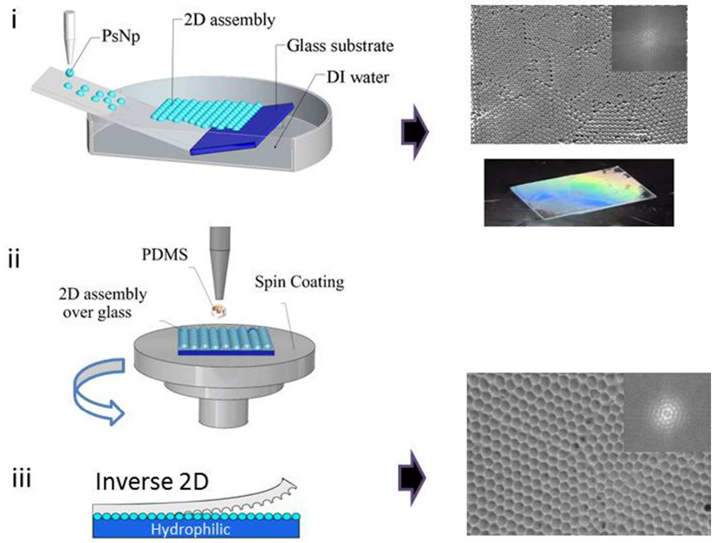

Fig. S1. Steps of the fabrication process.

2. Characterization of the polymeric nanostructure size.

The size of the voids leaved into the PDMS after the peeling process was measured from the SEM images by using ImageJ software. First, the SEM image was filtered to remove artifacts obtaining a clean image. After that, a threshold was applied in order to partitioning the image into a foreground and background defining the voids shapes, and the posterior representation of its outlines, as shown in Fig 2 ii). Second, a size distribution analysis of the cleaned image were performed, using the area covered by the voids and calculating the diameter size distribution represented by a histogram. Third, the histogram was fitted by a Gaussian curve, determining the average voids diameters, as shown in Fig 2 iii).

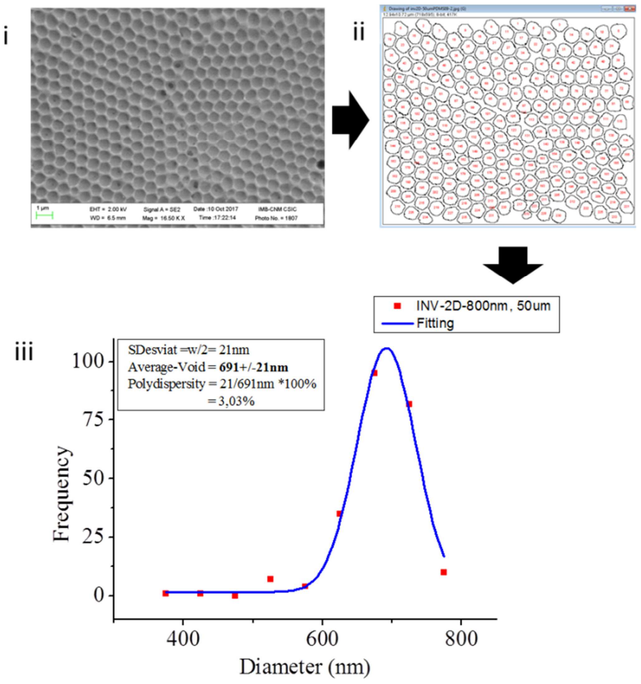

Fig. S2. Sequence of steps to analyze the voids size from the SEM images

3. Membrane sensitivity characterization: position dependency.

UV/Vis reflection spectrometry was used to characterize the color response of the photonic membranes by applying incremental constant pressures.

The color response of the membrane under pressure deformations were analyzed for a low pressure range of ± 1 kPa, focusing at different positions of the membrane. Fig. S3 shows a scheme of the positions of focus analysed, and the pressure-wavelength curves obtained in each case. The maximum wavelength change is different depending on the region of focus, being higher when focusing near the border of the membrane, in positions 1 and 5, where the angle changes are larger. The minimum induced wavelength change was measured when focusing at the center of the membrane. In this position, the angle contribution to the wavelength change is smaller, but still not negligible due to the size of the spot, and the contribution of the period change should be considered.

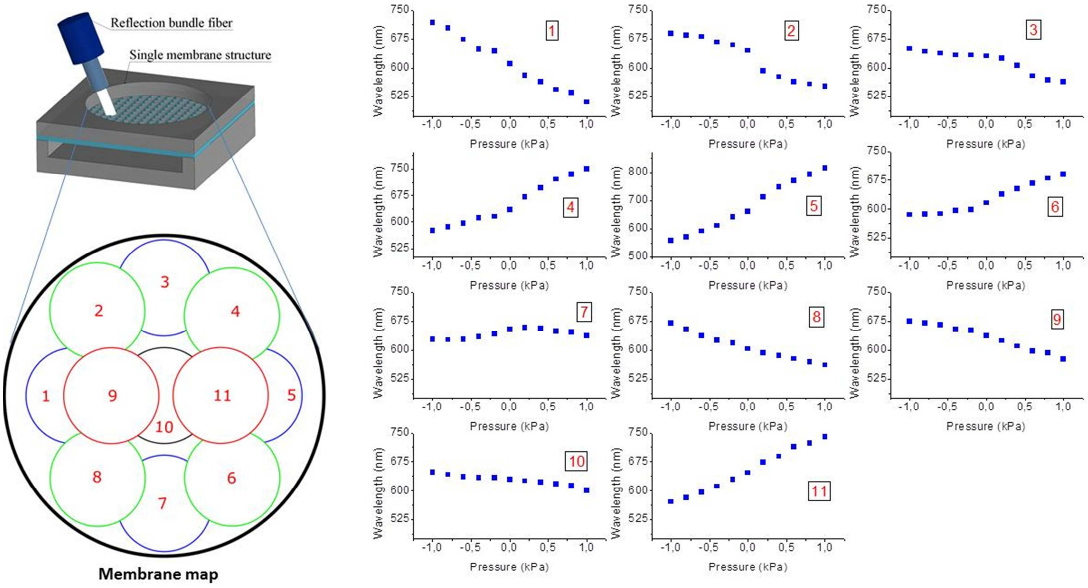

Fig. S3. UV-vis characterization curves performed at different positions of the membranes, for a range of pressure between -1 kPa and 1 kPa.

For a fixed position of the fibre, close to the border of the membrane, the signal shows a long-term stability (Fig. S4).

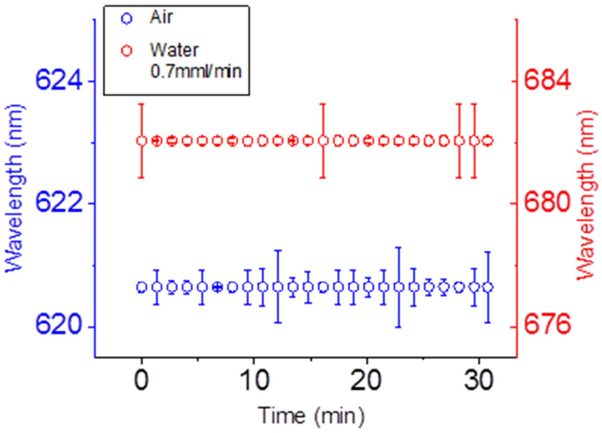

Fig. S4. Long-term stability of our signal (mean value of 3 different measurements in different days), both in air and liquid at 0.7 mml/min

4. Wavelength-angle dependency calibration curve

A calibration curve was obtained by measuring the color change produced when focusing in a flat nanostructured PDMS by changing the angle of incidence of the bundle reflection probe. In this case, a linear color change was found, as shown in Fig. S5. The experimental data were fitted to Eq.1 in Littrow configuration leaving the particle diameter as a variable parameter. The experimental results were fitted to the equation for a particle diameter of 790 nm, giving a wavelength change of around 20 nm per degree.

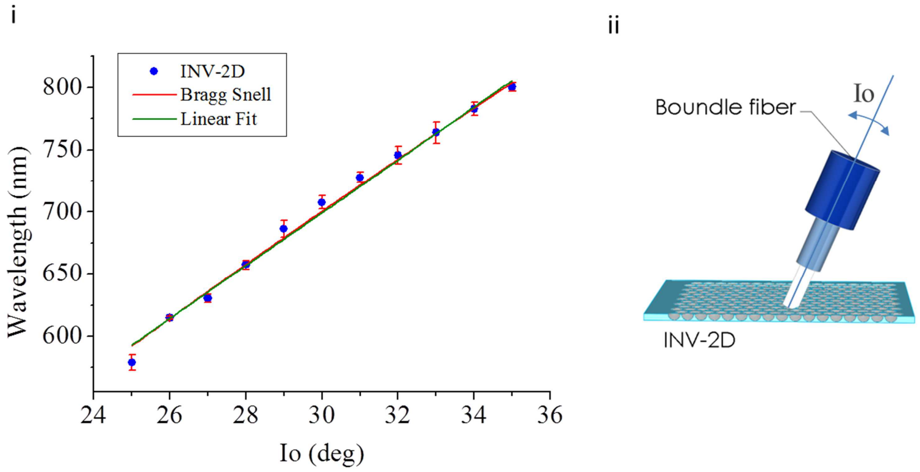

Fig. S5. Reflected wavelength-angle dependency calibration curve from a flat nanostructured PDMS (not suspended)
